# Genetically predicted the causal relationship between blood metal levels and ischemic stroke risk based on Asian ethnicity

**DOI:** 10.1097/MD.0000000000043898

**Published:** 2025-08-15

**Authors:** Zhaoyi Jing, Bingbing Wang, Wenzhu Hao, Xiao Ding

**Affiliations:** a The First Clinical Medical College, Shandong University of Traditional Chinese Medicine, Jinan, China; b Department of Neurology II, Shandong University of Traditional Chinese Medicine Affiliated Hospital, Jinan, China.

**Keywords:** Asian ethnicity, blood metal levels, genetically predicted, ischemic stroke, Mendelian randomization

## Abstract

Metal element supplementation leads to elevated concentrations of these minerals in the blood. Therefore, any association between blood metal levels and the risk of ischemic stroke is potentially of great public health significance. The results of many observational studies suggest a correlation between blood metal levels and ischemic stroke. However, observational study is influenced by confounding factors. Moreover, there were comparatively few studies on Asian populations. In this study, we aimed to investigate the causal relationship between blood metal levels and risk of ischemic stroke based on Asian ethnicity. We applied 2-sample Mendelian randomization to estimate the causal effects of 42 blood metal levels on ischemic stroke risk. We conducted scrutiny on the SNPs using the LDtrait online tool. A series of sensitivity analyses were conducted to examine the heterogeneity and pleiotropy of the results. We found that plasma calcium levels (β = 0.038, se = 0.016, *P* = .019) was a risk factor. Conversely, plasma chromium levels (β = −0.031, se = 0.015, *P* = .036) were shown to act as a protective factor for ischemic stroke. These findings were consistent across different sensitivity analyses. This research offers proof indicating a causal link between plasma concentrations of elemental calcium and chromium and an elevated risk of ischemic stroke among Asians. This highlights the significance of keeping track of blood metal concentrations as a possible preventive and intervention measure for ischemic stroke. Additional studies are necessary to confirm these results and investigate the biological mechanisms involved.

## 1. Introduction

Ischemic stroke, a common acute cerebrovascular disease, accounts for approximately 80% of all strokes.^[1]^ It is characterized by a high occurrence, significant disability rate, elevated mortality rate, and recurrent incidence, leading to a substantial burden on individuals, families, and society.^[2]^ Ischemic stroke has devastating consequences, including physical disability, cognitive impairment and even death.^[3]^ Initial medical intervention is of critical importance in managing ischemic stroke to reduce brain damage and improve prognosis. Risk factors for this condition encompass hypertension, diabetes, smoking, obesity, elevated cholesterol levels, and a sedentary lifestyle.^[4]^ Additionally, medical conditions like atrial fibrillation, carotid artery disease, and specific blood disorders can elevate the likelihood of experiencing an ischemic stroke.^[5]^ Ischemic stroke is a medical emergency, and early medical intervention is crucial for controlling brain damage and improving prognosis. The current main treatments and preventive measures include thrombolytic therapy, antiplatelet drugs, anticoagulant drugs, surgical interventions, and rehabilitation therapy.^[6]^

Metal elements play various important physiological roles in the human body, including catalyzing enzyme reactions, maintaining normal cellular function, and participating in cell signaling, among others.^[7]^ Some studies have suggested that blood metal levels are associated with the risk of ischemic stroke.^[8–10]^ Metal elements may affect the prognosis of ischemic stroke patients through various pathways, and early elevation of certain plasma metallic elements may indicate the risk of ischemic stroke. Therefore, the research is needed to establish a causal relationship between blood metal levels and ischemic stroke.

Observational studies often face challenges such as residual confounding, mediator over-adjustment, and reverse causality, which diminish their effectiveness in establishing causal relationships between exposures and outcomes.^[11]^ While randomized controlled trials (RCTs) can overcome these limitations, they may pose practical challenges.^[12]^ Mendelian randomization (MR) is an epidemiological research tool that assesses the causal association between an exposure factor and an outcome (e.g., disease occurrence or mortality) based on genetic variants related to the exposure factor. Like RCTs, MR studies “randomize” participants based on one or more genetic variants that influence the risk factor and attempt to determine whether carriers of these genetic variations have different disease risk compared to non-carriers. For this reason, MR is often referred to as “nature’s randomized controlled trial.” Traditional observational studies include 3 broad categories: cross-sectional studies, case–control studies, and cohort studies. The design of this type of study usually involves questionnaires, biomarker measurements, imaging techniques, bacterial cultures, and other techniques to obtain information related to the exposure. Exposures are analyzed statistically to determine if there is a correlation between exposure and outcome. In contrast, MR studies use genetic variants as instrumental variables (IVs) to assess the causal link between exposures and outcomes. Genetic variants are present from the time of birth. The existence of genes is stable. Therefore, there is no reverse causality in the associations obtained from MR studies, and the method is also effective against confounding factors.

To explore the causal link between BMLs and the prognosis of IS, we employed various MR methods, focusing on individuals of Asian ethnicity, and conducted several sensitivity analyses to ensure the robustness of the results. This approach offered valuable findings on the causative connection among metal levels in the blood and the prognosis of ischemic stroke, particularly in Asian communities.

## 2. Materials and methods

We conducted a bidirectional MR study to perform bulk analysis on the relationship between blood metal levels and ischemic stroke. No further ethical approval is needed as a result of the reanalysis of level data that was summarized previously. Moreover, our study adhered to the recommendations outlined in the strengthening the reporting of observational studies in epidemiology using MR (STROBE-MR) guidelines.^[13]^

MR analysis is based on the following 3 core assumptions: the selected IVs must be significantly associated with the exposure, the selected IVs should not have a direct effect on the outcome, but only influence the outcome through the specific exposure, and confounding factors are unrelated to the selected IVs.

**Figure 1.**
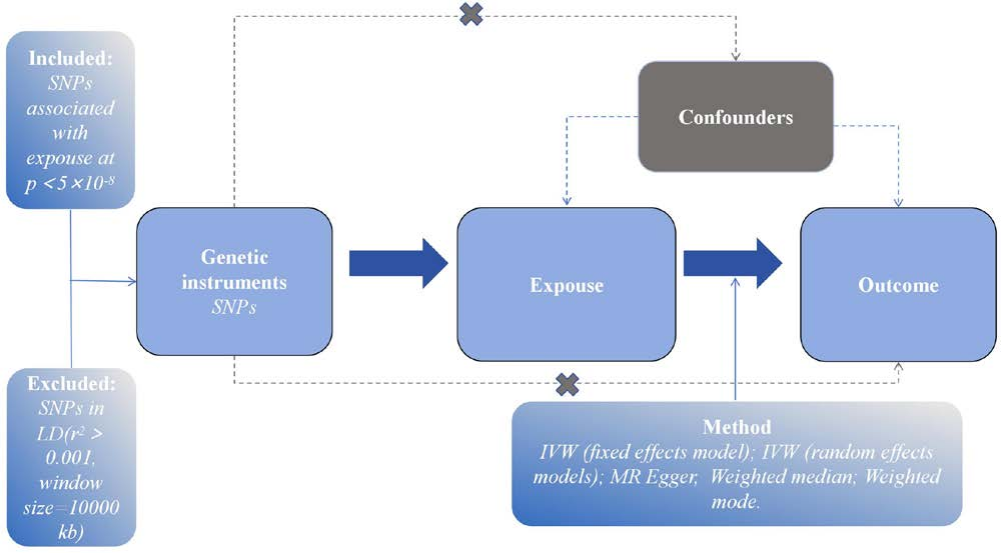
The overall design of Mendelian randomization analysis in the present study. Assumption 1, the genetic variants are supposed to be strongly associated with the risk of interest. Assumption 2, the genetic variants should not be associated with any confounding factors. Hypothesis 3, the genetic variants should affect the risk of the outcome only mediated by the exposures.

Due to the data being anonymized and publicly available, this study does not require approval from an ethics committee or informed consent from participants. The 3 main assumptions and specific details are illustrated in Figure 1.

1. ***GWAS data for blood metal levels***

The data on blood metal elements is derived from a genome-wide association study (GWAS).^[14]^ The study was based on data from the FAMHES (Fangchenggang Area Male Health and Examination Survey) and the MEC (manganese-exposed worker health cohort), both of which are population-based studies with Chinese ethnicity. And it included a total of 2488 participants. The GWAS data includes information on a total of 21 blood metal elements, including 42 sets of serum or plasma data. Further detailed information about the GWAS data can be found in the original research paper. In the primary analysis, a-value threshold of 5e−8 was applied to identify the cell factors with independent SNPs. Due to the limited number of cell factors reaching genome-wide significance, the *P*-value threshold was subsequently adjusted to 1e−5. To ensure no weak IVs were introduced, we calculated the F-values for each SNP. Please refer to Tables S1 to S4 (Supplemental Digital Content, https://links.lww.com/MD/P689) for details.

1. ***GWAS data for ischemic stroke***

To ensure population homogeneity and non-overlap of samples, the GWAS data for ischemic stroke also selected East Asian populations and came from different genome-wide association studies. Among them, the data from Japan with ID number bbj-a-129, includes 8,885,705 SNPs in total, 17,671 cases and 192,383 controls. Another set of GWAS data comes from a study of 220 deep-phenotype genome-wide association studies (disease, biomarkers, and drug use). This data also comes from Japan and was extracted from Biobank Japan, covering 12,457,433 SNPs, including 22,664 cases and 152,022 controls.^[15]^ Another set of ischemic stroke data (ID: ebi-eas-GCST90018644) originates from a 2021 GWAS conducted in Japan, involving a sample size of 174,686 participants. The dataset consists of 22,664 cases and 1,520,222 controls. Similarly, when conducting reverse MR analysis, we encountered the same issue where we could not obtain sufficient SNPs with a threshold of 5e−8. Therefore, we relaxed the threshold to 1e−5.

The GWAS data for exposures and outcomes are sourced from distinct databases. The characteristics of all the GWAS data used are detailed in Table S5 (Supplemental Digital Content, https://links.lww.com/MD/P689), Specifically, the blood metal element data comes from a Chinese database, while the ischemic stroke data comes from a Japanese one. By selecting data in this manner, we were able to ensure both non-overlapping samples and homogeneity of the study population in terms of ethnicity. Assessment methods and diagnostic criteria for ischemic stroke, detailed information can be found in the original paper.^[15]^

1. ***Statistical analysis***

To meet the requirements of assumption 1, this study initially set a stringent extraction threshold of *P* < 5e−08. However, given that an insufficient number of SNPs could be extracted under this threshold, it was appropriately relaxed to 1e−5.^[16]^ In order to eliminate linkage disequilibrium (LD) among IVs, a threshold of r² < 0.001 and a clump distance of > 10,000 kb were applied. After extracting SNP data for blood metal content, we first calculated the F-statistic for exposure using the formula (F = beta^2^/se^2^) to assess the strength of the IVs (F > 10 indicates sufficient strength).^[17,18]^ For assumptions 2 and 3, all included SNPs was screened with the LDtrait online tool (https://ldlink.nih.gov/?tab=ldtrait) to ensure that the 3 main assumptions presented by the study had been established.^[19]^ This rigorous screening process ensured that the selected IVs were free from confounding factors and that they solely influenced the outcome through the specified exposure. Additionally, the Steiger filtering method was used in this study to verify the correctness of the directional effects of individual SNPs. The bidirectional MR analyses were performed using the “TwoSampleMR” software package, and inverse-variance weighting (IVW) was used as the main determination method for the preliminary analyses. In addition, MR-Egger, weighted median, and weighted mode analyses were used to supplement the results. P-values of <0.05 were considered significant, and whether the exposure was a risk or protective factor was judged according to the β-value, and the results were further interpreted according to the OR. The IVW method assumes that all SNPs included in the analysis can be treated as valid IVs, at which point the method can provide greater assistance in the analysis.^[20]^ The weighted median gives an accurate estimate based on the assumption that the number of valid IVs is 50%.^[21]^ The MR-Egger regression assumes that all IVs are invalid IVs, and the estimation accuracy of this method is relatively low.^[22]^ Weighted mode are not as robust as IVW, but they provide a means of testing the consistency and stability of the results.^[23]^

## 3. Sensitivity analysis

In sensitivity analysis, we used Cochran *Q* test to assess heterogeneity.^[24]^ Heterogeneity primarily assesses variations among individual IVs; if these variations are significant, it suggests high heterogeneity. If heterogeneity was detected in our investigation, we used a random effects model in the IVW method, since random effects models provide more reliable results in the presence of heterogeneity than fixed effects models. Directional pleiotropy was verified using the MR-Egger intercept test^[22]^ and the MR-PRESSO test.^[25]^ In addition, we also employed the MR-PRESSO Outlier-corrected test to detect potential outliers that could introduce horizontal pleiotropy and provide corrected MR estimates after removing these outliers.^[25]^ Leave-one-out sensitivity analysis involves sequentially removing one IV and recalculating the MR analysis without it.^[26]^ If the MR results for the remaining IVs significantly differ from the overall estimate when an IV is removed, it suggests that the MR is sensitive to that IV. This method was employed in our sensitivity analysis to assess the robustness of our results. In addition, we employed the Steiger test for directionality to verify if our MR analysis results were consistent in the expected direction.^[27]^ One thing worth noting is that plasma levels of magnesium and aluminum were excluded from the sensitivity analysis due to a limited number of single nucleotide polymorphism (SNPs < 3). We also used Steiger filtering to assess the directional significance of individual SNP. If a SNP’s directionality was found to be incorrect, it would be removed from the analysis to maintain the integrity of the findings. This method is primarily employed to bolster the exclusivity assumption in the 3 fundamental hypotheses of MR studies.

The ratio of the odds ratio (OR) is used as the measure of effect, with a 95% confidence interval (CI) for each standard (SD) increase in risk exposure. A *P*-value less 0.05 is considered statistically significant. All tests are 2-tailed.

The analysis was conducted using the packages TwoSampleMR (version 0.5.6) and MR-PRESSO (version 1.0) in R software (version 4.4.2).

## 4. Results

1. *Causal effects of blood metal elements on ischemic stroke*

**Figure 2.**
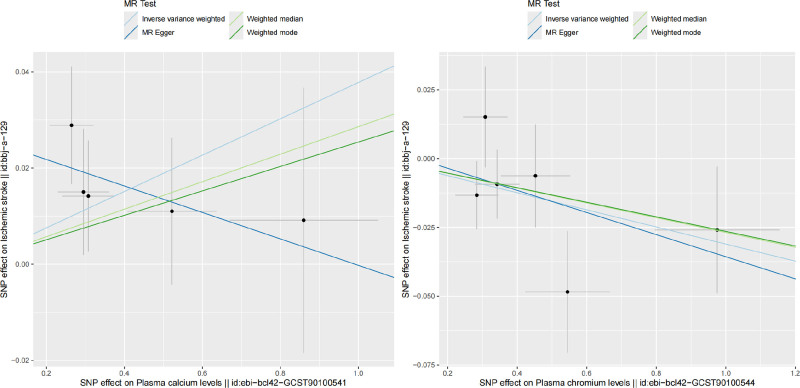
Scatter plots of MR analysis. MR = Mendelian randomization.

**Figure 3.**
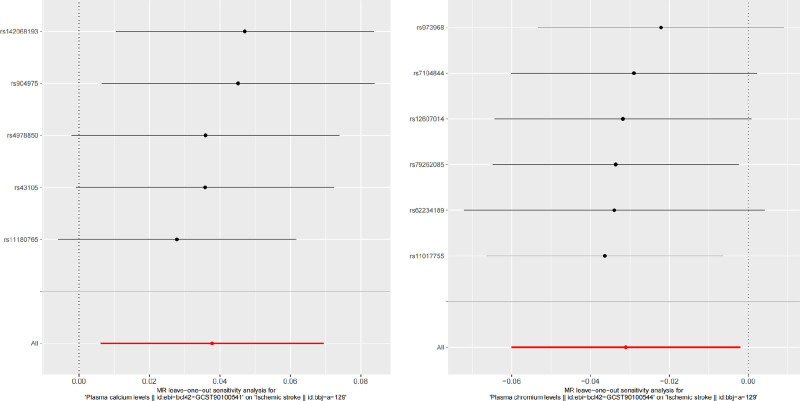
The results of leave-one-out analyses.

After removing SNPs associated with confounders and outcome, we found that when ischemic stroke GWAS data with ID number bbj-a-129 was used as the outcome, plasma calcium levels (β = 0.038, se = 0.016, *P* = .019) and plasma nickel levels (β = 0.033, se = 0.015, *P* = .025) were risk factors. Conversely, plasma chromium levels (β = −0.031, se = 0.015, *P* = .036) were shown to act as a protective factor for ischemic stroke. In an additional dataset, we uncovered the blood plasma nickel levels (β = 0.030, se = 0.013, *P* = .024) as a potential risk factor. The F-statistic values for all IVs are >10. In sensitivity analyses, Cochran *Q* test and MR-Egger intercept test did not reveal the presence of heterogeneity and pleiotropy, and MRPRESSO Outlier-corrected test did not detect the presence of outliers (Tables 1 and 2). However, it is worth noting that in the MRPRESSO test we found the presence of pleiotropy in the GWAS dataset of plasma nickel levels in both data sets. See detailed information about sensitivity analyses in Table 2. In Figure 2, a visually depicted Scatterplots illustrate the distribution of positive outcomes. Meanwhile, Figure 3 offers the result of leave-one-out methodology.

**Table 1:** Information of heterogeneity test.

Outcome	Exposure	Heterogeneity			
		Method	*Q*	*Q*_df	*Q*_pval
Ischemic stroke	Plasma calcium levels	MR-Egger	0.850	3.000	.837
		IVW	3.589	4.000	.464
Ischemic stroke	Plasma chromium levels	MR-Egger	4.148	4.000	.386
		IVW	4.230	5.000	.517

IVW = inverse-variance weighted, MR = Mendelian randomization.

**Table 2:** Information of pleiotropy.

Outcome	Exposure	MR-Egger intercept			MR-PRESSO				
		Egger intercept	SE	*P*-value	MR analysis	Causal estimate	SD	*T*-stat	*P*-value
Ischemic stroke	Plasma calcium levels	0.027	0.016	.197	Raw	0.038	0.015	2.472	.069
					Outlier-corrected	NA			
Ischemic stroke	Plasma chromium levels	0.004	0.016	.792	Raw	−0.031	0.014	−2.284	.071
					Outlier-corrected	NA			

MR-PRESSO = MR-pleiotropy residual sum and outlier, MR = Mendelian randomization, SD = standard deviation, SE = standard error.

1. *Causal effects of ischemic stroke on blood metal elements*

In analyzing the causal effect of ischemic stroke on blood metal element homeostasis, we found that ischemic stroke affected serum molybdenum levels (β = 0.235, se = 0.108, *P* = .029) and plasma cobalt levels (β = 0.463, se = 0.195, *P* = .018) only when the ischemic stroke (ID: bbj-a-129) GWAS dataset was used as the outcome. According to the formula, the F-statistic values for all SNPs are than 10. Therefore, all SNPs included in our analysis are strong instrumental. The Cochran *Q* test and the MR-Egger intercept test both failed to detect the presence of heterogeneity and pleiotropy, and the leave-one-out test did not find that a single SNP had a large effect on the outcome. However, the MRPRESSO test found the presence of pleiotropy, so we believe that the results are not statistically significant and robust.

**Figure 4.**
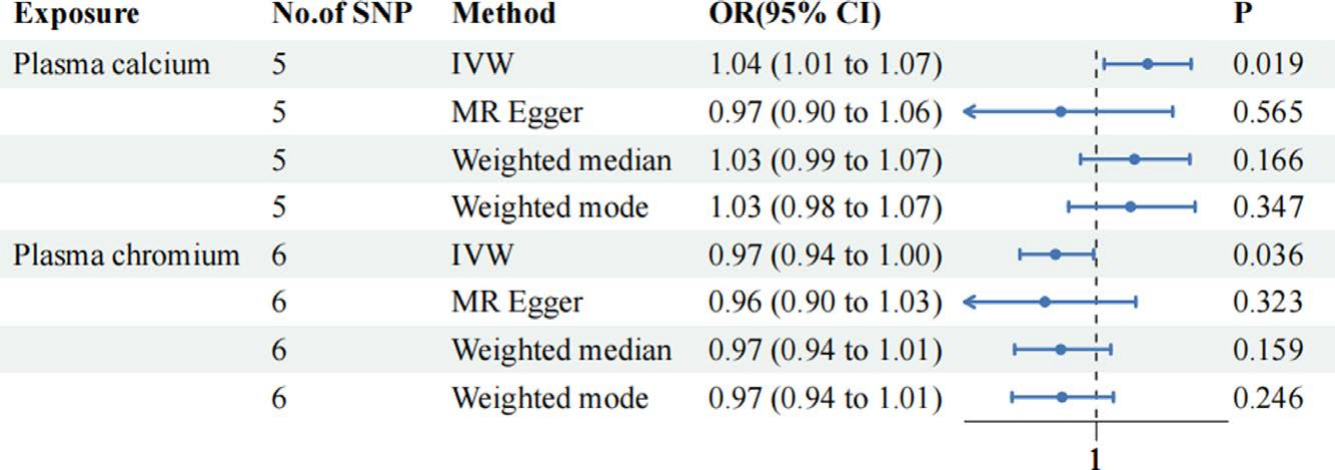
Forest plots of the causal effect of plasma calcium and chromium levels on ischemic stroke.

After a series of sensitivity analyses, we have finally determined the causal relationship between plasma calcium and plasma chromium levels and ischemic stroke. The forest plot is shown in Figure 4.

## 5. Discussion

Our MR study of 42 blood metal element levels found a positive association between plasma calcium and chromium levels and the risk of ischemic stroke, with robust results holding up in various sensitivity analyses. However, when ischemic stroke was considered as the exposure, we did not find any causal relationship between ischemic stroke and blood metal elements.

### 5.1. Comparison with previous studies

It is known that an increase in intracellular calcium levels is a major pathway leading to cell death in ischemic conditions. Previous research has already suggested an association between calcium and ischemic stroke. One of the earliest observational studies assessed the correlation between serum elemental calcium levels in patients with acute ischemic stroke by evaluating the calculation of infarct volume on diffusion-weighted magnetic resonance imaging.^[28]^ Although the study concluded that serum calcium was judged to be a valid clinical predictor, the study protocol was not designed to effectively control for confounding factors, so the results may be biased. Unlike the previous study, Ovbiagele B and his team employed a research design called repinotan–randomized exposure controlled trial (mRECT) to investigate the association between serum calcium levels and ischemic stroke.^[29]^ In the study, they utilized more commonly used assessment measures such as the median NIHSS (National Institutes of Health Stroke Scale) score, median barthel index score, and modified Rankin Scale Score to evaluate the physical condition of participants. The research effectively controlled the baseline and used a variety of statistical methods to assess possible confounders. It greatly improved the credibility of the study’s findings. In the end, the trial discovered a prognostic role for serum calcium (Ca2+) levels between 72 and 96 hours, while no correlation was found between early serum calcium levels and the prognosis of patients with ischemic stroke. Later, in a follow-up study, Appel et al made a new discovery.^[30]^ In the process of constructing their model, they found the association between serum calcium concentration and mortality rate in acute stroke patients was nonlinear. In the same year, a Chinese study focusing on nutrition yielded contrasting results. Liang et al discovered that calcium supplementation helped reduce the risk of ischemic stroke in individuals from southern China.^[31]^ We speculate that the inconsistency in results may be attributed to differences in race or ethnicity. It is also possible that the study did not examine the distribution of calcium within the body, leading to differences in research outcomes. Therefore, the distribution of calcium within the body and its impact on individuals of different races or ethnicities should be taken into consideration. Similarly, research from India has also found a significant negative correlation between total calcium and ionized calcium levels with IS.^[32]^ The following year, a prospective study found that lower calcium levels upon admission in acute stroke patients worsened their clinical symptoms.^[33]^ It is worth mentioning that, in studies examining the association between serum calcium levels and ischemic stroke, this study introduced the modified Rankin Scale (mRS) score for evaluating the condition of ischemic stroke patients for the first time. However, the study had a relatively small sample size, which limits its power of persuasion. Building upon previous findings, a meta-analysis was conducted to analyze the association between circulating calcium levels and cerebrovascular disease.^[34]^ This analysis also confirmed that circulating calcium levels are indeed a risk factor for cerebrovascular disease. Similarly, a subsequent prospective study, conducted by Chung et al, reported the same finding, suggesting that elevated albumin-corrected serum calcium levels were linked to a worse short-term prognosis and a heightened long-term risk of death following an acute ischemic stroke.^[35]^ In line with studies conducted in Asia, a REGARDS study also discovered that elevated serum calcium levels were associated with a reduced risk of ischemic stroke.^[36]^ Furthermore, this study identified a threshold effect of serum calcium levels on the risk of ischemic stroke. After including 9375 patients with ischemic stroke, Zhang et al affirmed the prognostic value of serum calcium levels through data analysis.^[37]^ Serum calcium levels may serve as a potential therapeutic target for targeted interventions. A study from the MIMIC database, for the first time, analyzed calcium elements by dividing them into serum total calcium (tCa) and serum ionized calcium (iCa).^[38]^ It concluded that both upregulation and downregulation of tCa were associated with poor short-term prognosis. Therefore, from this study, it appears that blood calcium homeostasis is particularly important for the prognosis of ischemic stroke patients. Finally, some clinical studies on the effects of calcium channel blockers in the treatment of ischemic stroke indirectly suggest a correlation between intracellular calcium levels and ischemic stroke.^[39]^

There have been previous MR studies that analyzed the relationship between serum calcium levels and ischemic stroke.^[40,41]^ However, these studies did not establish a causal relationship between plasma calcium levels and ischemic stroke, and the studies primarily focused on European populations as study subjects. This may have contributed to the inconsistencies in the results.

There is relatively limited observational research regarding the association between blood chromium levels and ischemic stroke. In an animal experiment, it was found that chromium supplementation can improve the prognosis of patients with ischemic stroke.^[42]^ They believe that chromium mainly achieves this by improving hyperglycemia, reducing plasma insulin and corticosterone levels, and decreasing the size of cerebral infarction. In addition, a study on the impact of air pollution on the incidence of ischemic stroke also discovered a correlation between exposure to chromium elements and ischemic stroke.^[43]^

The inconsistency among different studies can be explained by the differences in study design and measurement of outcomes. In our study, we employed MR analysis to mitigate the biases commonly observed in observational studies and utilized data from a large-scale genetic consortium.

1. *Possible explanations*

In our MR study, we did not find a causal relationship between serum calcium levels and ischemic stroke. However, a positive association was observed between plasma calcium levels and ischemic stroke (*P* <  .05).

First, the concentration of cyclic calcium ions can affect the calcium ion homeostasis within the cell, which is closely related to cellular apoptosis. Calcium ions disrupt the mitochondria, causing mitochondrial swelling and leading to the loss of integrity of the outer mitochondrial membrane.^[44]^ This, in turn, triggers the release of apoptotic factors such as mitochondrial apoptosis-inducing factor 1 and endonuclease G, initiating the intrinsic pathway of cell apoptosis. Eventually, this cascade of events culminates in cellular demise.

Second, the accumulation of calcium has been proven to cause mitochondrial damage. Mitochondrial dysfunction is an important factor influencing the prognosis of ischemic stroke.^[45]^ Therefore, we believe that mitochondrial dysfunction caused by this accumulation is one of the important mechanisms by which increased plasma calcium levels increase the risk of ischemic stroke.^[46]^ The combination of oxidative stress and mitochondrial calcium accumulation can lead to depolarization uncoupling, resulting in the generation of reactive oxygen species (ROS). ROS are byproducts of normal oxygen metabolism.^[47,48]^ Under normal circumstances, oxygen participates in oxidative phosphorylation and is reduced to water (H_2_O). However, under certain pathological conditions, electrons may leak prematurely in the electron transport chain, reacting with oxygen to generate superoxide anions, which are known as ROS.^[48]^ Due to their strong oxidative properties, ROS can cause oxidative damage to proteins, lipids, and nucleic acids within cells, leading to cellular dysfunction.^[49]^ Excessive ROS can trigger inflammatory responses, resulting in chronic inflammation, which has been linked to the development of various diseases.^[50]^ The continuous increase in ROS levels is harmful to mitochondria, and mitochondrial damage further promotes the generation of ROS, ultimately leading to more widespread damage. The excessive production of ROS can contribute to widespread neuronal damage and death, leading to poor prognosis in ischemic stroke.^[51]^

In addition, calpain, a calcium-dependent protease, is also recognized as an important mechanism that could influence the prognosis of ischemic stroke.^[52]^ This protease is capable of degrading crucial proteins in neurons in a calcium ion-dependent manner, ultimately leading to neuronal death.

Lastly, calcium ions are crucial clotting factors. Although only a minute quantity of calcium ions is required for blood coagulation, they are essential constituents in almost all stages of the clotting process.^[53]^ The normality of the blood clotting system is closely related to stroke prognosis.^[54]^ Additionally, we believe that one of the differences between serum and plasma is that serum lacks fibrinogen, which may be a reason for the inconsistency in calcium levels between serum and plasma.

We have also discovered a causal relationship between plasma chromium levels and ischemic stroke. Chromium element may influence the prognosis of ischemic stroke through multiple mechanisms. In its trivalent form, chromium is crucial for mammals as it plays a pivotal role in ensuring appropriate metabolism of carbohydrates and lipids.^[55,56]^ First, several studies have found chromium to have positive effects in the brain, improving cognitive function as well as reducing depressive symptoms in older adults.^[57,58]^ This suggests that chromium may have some neuroprotective effects. Second, trivalent chromium also participates in blood glucose regulation by acting as a second messenger promoting insulin neuromodulation by amplifying insulin signaling.^[59]^ Influencing prognosis by regulating blood glucose may be another important mechanism by which plasma chromium affects ischemic stroke. Lastly, chromium supplementation also possesses anti-inflammatory and antioxidant effects, particularly in regulating insulin-like growth factor 1 signaling.^[60,61]^ Nezami et al found that patients with cerebrovascular accident had significantly higher levels of copper and lead and significantly lower levels of nickel.^[62]^ However, the present study did not find an association between these 3 circulating metallic elements and ischemic stroke, which may be the result of potential confounding factors interfering.

More research is needed to further validate the pathological mechanisms of the influence of plasma chromium and plasma calcium on ischemic stroke.

1. *Strengths and limitations*

First, a major strength of this study lies in the application of MR methodology. In MR analysis, we grabbed genetic data from large-scale GWAS data based on a large sample size, thus improving the accuracy of SNP selection and the statistical power of analysis. Compared to traditional observational studies, this study effectively addresses biases arising from confounding factors and reverse causation. Second, we employed a variety of MR analyses to complement the IVW method. A series of sensitivity analyses were also conducted to effectively control for bias due to pleiotropy and to effectively address heterogeneity through random effects modeling. Finally, we examined the LD of the included genetic variants.

However, it is important to recognize the limitations of this research and interpret the findings with caution. The level of evidence-based medical evidence for MR studies is properly recognized.^[63]^ First, despite the use of multiple magnetic resonance methods to prevent confounding pleiotropy, residual bias could not be eliminated because of the inherent limitations of MR studies. For example, it is not possible to completely rule out the possibility of blood metal-related SNPs affecting ischemic stroke through alternative causal pathways rather than through blood metal exposure. We were also unable to measure threshold effects for exposures for which a causal relationship existed. Second, because of data limitations, we were unable to analyze subtypes of ischemic stroke or to analyze blood metal elements in different states (e.g., divalent vs trivalent iron). Therefore, we could not determine whether there were differences in the effects of blood metal elements on different ischemic stroke subtypes. It was also unable to assess whether there were differences in the causal relationship between different forms of blood metal elements and ischemic stroke. Furthermore, the proportion of variation in blood metal elements explained by genetic variants is relatively low. Although no causal relationship was observed between the levels of other blood metal elements and ischemic stroke, we cannot completely rule out the possibility that our study may not have had sufficient power to detect weak associations. Lastly, it is important to note that our study was conducted with a specific ethnic group (Asian population), which may limit the generalizability of the findings to other racial/ethnic groups.

We should also have a correct perspective on the relationship between randomized controlled trials (RCTs) and MR studies. Although RCTs may have limitations such as heterogeneity between study samples and real-world populations due to inclusion/exclusion criteria, as well as constraints related to ethics, participant compliance, and study duration, they remain the gold standard for evaluating causal effects. Therefore, we should cautiously interpret the results of these studies.

1. *Clinical and research implications*

Our research findings suggest a positive correlation between predicted plasma calcium levels and the risk ischemic stroke, while plasma chromium levels to be protective factor against ischemic stroke. This supports the reasonable speculation that downregulating plasma calcium and upregulating serum chromium may help reduce the risk of ischemic stroke. It provides genetic-level recommendations for clinical nutrition therapy. Additionally, plasma levels of calcium and chromium elements may serve as indicators for clinical prognosis in evaluating the outcome of ischemic stroke patients. Finally, our study provides recommendations for public health management, suggesting preventive interventions to reduce exposure to hazardous metal elements among populations with risk factors for ischemic stroke. Although MR can establish causality, it does not explain the complex mechanisms linking exposure and outcomes. Therefore, future comprehensive studies are needed to confirm our findings, elucidate their underlying biological mechanisms, develop appropriate monitoring, treatment, and prevention strategies and managing nutrition in individuals at risk of ischemic stroke.

## 6. Conclusion

This is the first comprehensive 2-sample MR study based on Asian population, examining the potential associations between genetic predictions of 21 serum or plasma metal elements and the risk of ischemic stroke. Through our analysis, we observed a causal relationship for plasma calcium and chromium levels with ischemic stroke. These 2 positive findings were robust across different sensitivity analyses. However, more investigations are necessary to examine the robustness of our results and to further characterize the underlying mechanisms.

## Author contributions

**Conceptualization:** Zhaoyi Jing, Xiao Ding.

**Data curation:** Zhaoyi Jing, Xiao Ding.

**Formal analysis:** Wenzhu Hao, Xiao Ding.

**Funding acquisition:** Wenzhu Hao, Xiao Ding.

**Investigation:** Zhaoyi Jing.

**Methodology:** Zhaoyi Jing.

**Project administration:** Zhaoyi Jing.

**Resources:** Zhaoyi Jing.

**Software:** Bingbing Wang, Wenzhu Hao.

**Supervision:** Bingbing Wang, Wenzhu Hao.

**Validation:** Bingbing Wang, Wenzhu Hao.

**Visualization:** Bingbing Wang, Wenzhu Hao.

**Writing – original draft:** Zhaoyi Jing.

**Writing – review & editing:** Wenzhu Hao, Xiao Ding.

## Supplementary Material


